# Free Triiodothyronine Is Associated with Poor Outcomes after Acute Ischemic Stroke

**DOI:** 10.1155/2022/1982193

**Published:** 2022-02-03

**Authors:** Yue Song, Changqiang Yang, Hua Wang

**Affiliations:** ^1^Department of Pediatrics, West China Second University Hospital, Sichuan University, Chengdu, China; ^2^Key Laboratory of Birth Defects and Related Diseases of Women and Children (Sichuan University), Ministry of Education, Chengdu, China; ^3^Department of Cardiovascular Medicine, West China Hospital, Sichuan University, Chengdu, Sichuan, China

## Abstract

**Aims:**

It is unclear whether thyroid hormones are associated with functional outcomes after ischemic stroke. We aimed to investigate the impact of serum levels of thyroid hormones at admission on functional outcomes at 3 months after acute ischemic stroke.

**Methods:**

A total of 480 consecutive patients with ischemic stroke who were admitted to our hospital within 48 h of onset were enrolled. The levels of thyroid hormones, including thyroid-stimulating hormone, free triiodothyronine (FT3), and free thyroxine, were measured at admission, and functional outcomes were assessed at 3 months using the modified Rankin Scale (mRS), with scores ranging from 0 to 6. Poor outcome was defined as mRS score ≥3.

**Results:**

FT3 levels at admission were considerably lower in patients with poor outcomes than in those with good outcomes at 3 months (3.53 ± 0.70 pmol/L vs. 4.04 ± 0.68 pmol/L; *P* < 0.001). Lower FT3 levels were observed in patients with higher mRS scores. Multivariable logistic regression analysis revealed that FT3 levels were significantly associated with a risk of poor outcomes at 3 months, independent of conventional risk factors such as age, National Institutes of Health Stroke Scale score, and recanalized therapy. In addition, patients in FT3 levels in the lowest quartile had a 2.56-fold higher risk of poor outcomes than those with FT3 levels in the highest quartile (odds ratio = 2.56, 95% confidence interval = 1.15–5.69, *P*=0.021). The sensitivity and specificity of FT3 level ≤3.69 pmol/L for predicting poor outcomes were 62.70% and 72.03%, respectively.

**Conclusion:**

Our study suggests that FT3 levels at admission are significantly and independently associated with a risk of poor outcomes after ischemic stroke and that lower FT3 levels can be considered as a prognostic biomarker for poor outcomes at 3 months.

## 1. Introduction

In China, the burden of stroke is very high, and the annual stroke mortality rate is approximately 1.6 million, which has exceeded that of heart disease, becoming the leading cause of death and adult disability [1]. Among all types of stroke, ischemic stroke is the most prevalent, accounting for 69.6% cases [2]. With government financial support, the prognosis of Chinese stroke patients appears to have improved and is not very bad [3, 4]. Currently, the severity of ischemic stroke and functional outcomes are assessed using standardized clinical criteria [5, 6]. However, the prognostic value of these clinical parameters in ischemic stroke is subjective and insufficient. Therefore, identification of new biomarkers may potentially improve the accuracy of the current prognostic scales for functional outcomes.

Recent studies have suggested that serum levels of thyroid hormones are altered in the acute phase of ischemic stroke, which may have a potential influence on functional outcomes [7, 8]. Some studies have suggested that thyroid hormones play a neuroprotective role in recovery after ischemic stroke [9] and show a protective association between subclinical hypothyroidism and better outcomes [10, 11]. However, other studies have suggested that lower serum T3 levels are associated with poor outcomes after ischemic stroke [8, 12]. In addition, it is unclear whether there is any association between free triiodothyronine (FT3), the bioactive form of T3, and functional outcomes after ischemic stroke. Studies on the relationship between FT3 levels and functional outcomes after ischemic stroke have reported controversial results [12–18]. Therefore, we aimed to investigate whether serum levels of thyroid hormones, including thyroid-stimulating hormone (TSH), FT3, and free thyroxine (FT4), at admission are associated with functional outcomes at 3 months after acute ischemic stroke.

## 2. Materials and Methods

### 2.1. Study Population

This single-center, retrospective, observational study was performed at West China Hospital, Sichuan University, in accordance with the Declaration of Helsinki, with a waiver of ethics application due to its retrospective nature and minimal risk to patients. Informed consent was acquired with oral through telephone in all patients. This study enrolled patients with acute ischemic stroke admitted to our hospital between May 1, 2018, and October 1, 2019. The inclusion criteria for the patients were as follows: (1)age ≥18 years, (2)ischemic stroke confirmed by computed tomography (CT) or magnetic resonance imaging (MRI) at admission, and (3) ischemic stroke onset within 48 hours before admission. The exclusion criteria were as follows: (1)history of thyroid diseases, such as hyperthyroidism or hypothyroidism and (2) acute hemorrhagic stroke and transient ischemic stroke. The management of all patients with acute ischemic stroke was based on the Chinese guidelines for the diagnosis and treatment of acute ischemic stroke [19].

### 2.2. Data Collection

Data on baseline characteristics, such as demographic features (age and sex), risk factors (hypertension, diabetes mellitus, atrial fibrillation, smoking, and excessive alcohol consumption), history of concomitant cardiovascular diseases (coronary heart disease and heart failure), and stroke, were collected at admission. Anthropometric parameters (weight and height) and blood pressure were also measured at admission. The severity of stroke at admission was assessed using the National Institutes of Health Stroke Scale (NIHSS) [5]. Routine laboratory investigations, including measurement of platelet, white blood cell count, hemoglobin, glucose, albumin, estimated glomerular filtration rate, uric acid, triglyceride, total cholesterol, and high-density lipoprotein cholesterol levels, were also performed. In addition, serum levels of TSH, FT3, and FT4 were measured the following morning. The normal values for TSH, FT3, and FT4 are 0.27–4.2 mIU/L, 3.6–7.5 pmol/L, and 12–22 pmol/L, respectively. All measurements were performed by laboratory staff who were blinded to the clinical information. All patients underwent head CT or MRI at admission. The subtype of stroke was determined according to the Trial of Org 10172 in the Acute Stroke Treatment (TOAST) subtype classiﬁcation system [20]. We classified ischemic stroke into the following subtypes: large-artery atherosclerosis, cardioembolism, small-artery occlusion, and unclassified (others or undetermined etiology).

### 2.3. Endpoints

Functional outcomes of all patients were assessed using the modified Rankin Scale (mRS) at 3 months after ischemic stroke, with scores ranging from 0 (no symptoms) to 6 (death) [21]. Information on functional outcomes was collected via direct or telephone interviews with patients or family members by trained neurologists. mRS scores of 0–2 indicated good outcomes, and mRS scores of 3–6 indicated poor outcomes [22].

### 2.4. Statistical Analysis

All data were analyzed using IBM SPSS Statistics version 21 (IBM Corp., Armonk, NY). Normally distributed continuous data were presented as means ± standard deviations. Data with a skewed distribution were expressed as medians (quartiles). Categorical variables were expressed as percentages. The baseline characteristics of the groups were compared using Student's *t*-test, the Mann–Whitney *U* test, or the chi-square test, depending on the appropriate value properties. Logistic regression analysis was used for correlation analysis. The results of poor outcomes at 3 months were presented as odds ratios (ORs) and 95% confidence intervals (CIs). The area under the curve (AUC) of the receiver operating characteristic (ROC) curve was calculated using the predicted probability of poor outcomes. The results were considered significant at *P* < 0.05.

## 3. Results

**Figure 1:**
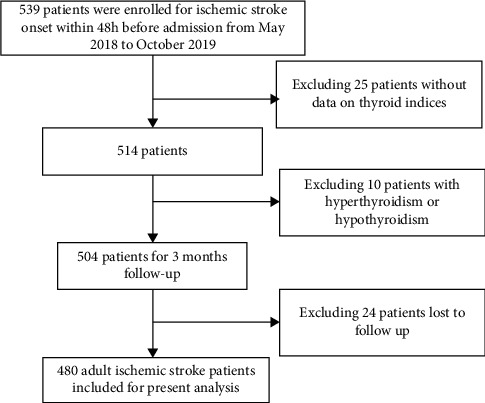
Study flow diagram.

Among the 539 patients, 25 patients without data on thyroid indices, 10 patients diagnosed with hyperthyroidism or hypothyroidism, and 24 patients who were lost to follow-up were excluded. Finally, 480 patients with ischemic stroke were included (Figure 1). During the 3-month follow-up, 244 (50.8%) patients had poor outcomes. The baseline characteristics (demographic, clinical, and laboratory data) of the patients according to the functional outcomes assessed by the mRS score are presented in Table 1. Compared to patients with good outcomes, those with poor outcomes were older, had lower hemoglobin and albumin levels and estimated glomerular filtration rate (eGFR), and had higher white blood cell count and NIHSS scores. The proportion of women (*P*=0.001), patients with atrial fibrillation (*P* < 0.001) and coronary heart disease/heart failure (*P*=0.001), and patients receiving recanalized therapy (*P*=0.005) were also significantly higher in the poor outcome group than in the good outcome group. In addition, patients with poor outcomes had lower TSH and FT3 levels, but higher FT4 levels, than those with good outcomes.

To explore the independent risk factors for poor outcomes at 3 months, we used variables with *P* values <0.1 in univariate analysis in a multivariable logistic regression analysis. The results revealed that lower FT3 level was an independent risk factor for poor outcomes at 3 months (OR = 0.561; 95% CI = 0.375–0.841, *P*=0.005). In addition, older age (OR = 1.023, 95% CI = 1.001–1.047, *P*=0.044), higher NIHSS score (OR = 1.286, 95% CI = 1.213–1.363, *P* < 0.001), and no recanalization therapy (OR = 3.527, 95% CI = 1.808–6.880, *P*=0.002) were also independent risk factors for poor outcomes. However, TSH and FT4 levels were not statistically significant after adjusting for other variables (Table 2).

**Figure 2:**
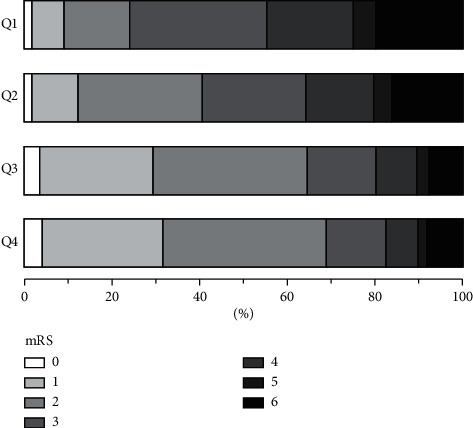
Distribution of mRS scores at 3 months according to the FT3 quartile.

**Figure 3:**
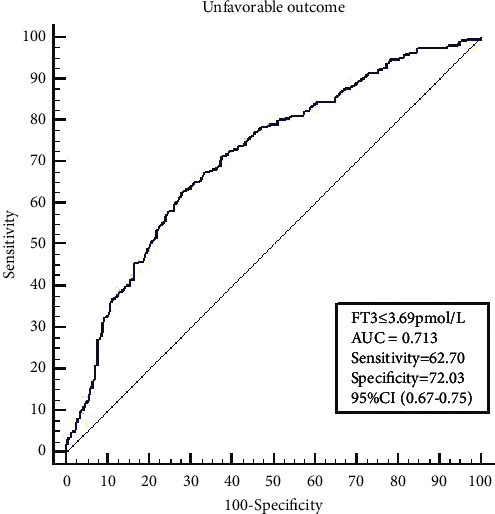
Receiver operating characteristic analysis of FT3 for the prediction of 3-month poor outcomes after ischemic stroke.

Patients were divided into quartile groups according to their FT3 levels. The distribution of mRS scores at 3 months differed according to the FT3 quartile (Q1, Q2, Q3, and Q4) (Figure 2); patients with higher mRS scores had lower FT3 levels. Further analyses were performed by constructing two models to explore the relationship between FT3 levels and poor outcomes. In model 1, adjusted for sex, atrial fibrillation, coronary heart disease and heart failure, hemoglobin level, white blood cell count, albumin level, eGFR, TSH level, triglyceride level, total cholesterol level, FT4 level, and TOAST classification, lower FT3 levels were associated with a worse functional outcome at 3 months (OR = 4.63, 95% CI = 2.33–9.02, *P* < 0.001 for Q1 vs. Q4 and OR = 2.42, 95% CI = 1.35–4.35, *P*=0.003 for Q2 vs. Q4). In model 2, additionally adjusted for age, NIHSS score, and recanalized therapy, FT3 levels were still signiﬁcantly associated with poor outcomes (OR = 2.56; 95% CI = 1.15–5.69, *P*=0.021 for Q1 vs. Q4) (Table 3). Additionally, the ROC curve analysis (Figure 3) revealed that a serum FT3 level ≤3.69 pmol/L was a powerful predictor of poor outcomes in patients with acute ischemic stroke (sensitivity = 62.70%; specificity = 72.03%; AUC = 0.713).

## 4. Discussion

Our study showed that lower FT3 levels were associated with poor outcomes at 3 months after acute ischemic stroke and that this association remained significant after adjusting for the effect of risk factors and comorbidities. The findings of two previous studies were consistent with our ﬁndings. Ambrosius et al. [18] found that, in patients with acute ischemic stroke, lower free T3 levels were an important factor related to unfavorable outcomes. Zhang et al. [16] found that FT3 levels significantly decreased in patients with poor outcomes after acute ischemic stroke and served as an independent predictor for neurological outcomes; the cutoff value of the FT3 level was 4.38 pmol/L. However, O'Keefe et al. [13] enrolled 129 patients with ischemic stroke and found that low FT3 levels were not associated with poor outcomes at 3 months in multivariate analysis when other known predictors of outcome, such as the severity of stroke (NIHSS score), were controlled. These contradictory findings could be due to the relatively small sample size of the study. In addition, our study also showed that the detrimental effect of FT3 levels on the functional outcome increased as FT3 levels decreased and that neither TSH nor FT4 levels were associated with poor outcomes at 3 months after ischemic stroke. One previous study conﬁrmed and strengthened our ﬁndings. Suda et al. [23] retrospectively enrolled 398 consecutive patients with ischemic stroke to investigate the association between serum thyroid hormone levels at admission and functional outcomes, showing a dose-effect relationship between FT3 levels at admission and poor outcomes. In addition, critical illness could result in decreased peripheral conversion of FT4 to FT3 in the absence of a primary thyroid disorder, indicating that FT3 might be more sensitive to the influence of critical illness than FT4 and TSH [24], which indirectly supported our findings.

The potential pathophysiological mechanism linking low FT3 levels to poor outcomes has not been well established. Basic research has indicated that the thyroid hormone T3 can cross the blood-brain barrier and promote neuronal development by improving the astrocytic microenvironment [25]. In addition, T3 plays a role in neuroprotection under ischemic conditions by reducing extracellular glutamate toxicity [25] and increasing the density of the brain vasculature [26]. Clinical studies have also shown that low T3 syndrome is independently associated with a risk of hemorrhagic transformation in acute ischemic stroke [27]. Low FT3 levels at admission increase the risk of poststroke infection and may contribute to poor outcomes [28]. Therefore, lower T3 levels have a detrimental effect on the outcome of ischemic stroke. Although it is unknown whether administration of exogenous thyroid hormones in patients with ischemic stroke improves outcomes, an animal experiment has demonstrated that administration of T3 can modulate neuronal plasticity mechanisms to enhance functional outcomes after stroke [29]. Therefore, further well-designed studies and randomized controlled trials are needed to elucidate the mechanism underlying the association between FT3 levels and functional outcomes and determine whether T3 supplementation can reduce disability after acute ischemic stroke, but our study indicates that FT3 levels can be regarded as a new biomarker to predict functional outcomes after acute ischemic stroke to some extent.

Several limitations of this study should be considered when interpreting our study. First, the study was based on single-center data, inevitably leading to selection bias. Second, we only measured thyroid hormone levels once and not dynamically as a function of time. Third, we did not have the opportunity to measure total T3 and T4 levels, which might have provided slightly different results.

## 5. Conclusion

Our data suggest that low FT3 levels at admission are independently associated with poor outcomes in patients with acute ischemic stroke after 3 months. Further investigations are needed to explore the mechanisms underlying this association and their potential exploitation in terms of therapeutic strategies to reduce disability after acute ischemic stroke.

## Figures and Tables

**Table 1:** Baseline characteristics (demographic, clinical, and laboratory data) according to the functional status at 3 months.

Variable	Good outcome (*n* = 236)	Poor outcome (*n* = 244)	*P* value
Age (years)	64.53 ± 14.22	70.11 ± 12.02	<0.001
Women (*n*, %)	69 (29.2)	107 (43.8)	0.001
BMI (kg/m^2^)	23.5 ± 3.2	23.1 ± 3.2	0.213
Smoking (*n*, %)	78 (33.0)	76 (31.1)	0.655
Alcohol consumption (*n*, %)	64 (27.1)	57 (23.3)	0.343
History of stroke (*n*, %)	23 (9.7)	26 (10.6)	0.742
Hypertension (*n*, %)	131 (55.5)	144 (59.0)	0.437
AF (*n*, %)	50 (21.1)	101 (41.4)	<0.001
DM (*n*, %)	60 (25.4)	64 (26.2	0.840
CHD/HF (*n*, %)	47 (19.9)	81 (33.2)	0.001
SBP at admission	140.58 ± 25.82	142.27 ± 22.76	0.449
DBP at admission	81.86 ± 15.78	80.40 ± 14.53	0.293
Hemoglobin (g/L)	139.09 ± 17.87	134.14 ± 20.15	0.005
Platelet (10^9/L)	181.20 ± 60.95	173.41 ± 66.84	0.183
White blood cell (10^9/L)	7.63 ± 2.75	8.48 ± 3.16	0.002
Glu (mmol/L)	7.97 ± 3.31	8.27 ± 3.30	0.316
Albumin (g/L)	42.41 ± 4.15	40.70 ± 4.09	<0.001
eGFR (ml/min/1.73 m^2^)	85.12 ± 19.29	79.69 ± 21.62	0.004
Uric acid (*μ*mol/L)	363.86 ± 106.12	349.23 ± 106.93	0.162
TG (mmol/L)	1.35 (0.93–2.05)	1.23 (0.88–1.73)	0.05
TC (mmol/L)	4.40 ± 1.04	4.26 ± 0.91	0.055
HDL (mmol/L)	1.24 ± 0.35	1.27 ± 0.37	0.334
NIHSS score	3 (2–7)	13 (8–17)	<0.001
TOAST classification			
LAA (*n*, %)	65 (27.5)	94 (38.5)	<0.001
CE (*n,* %)	51 (21.6)	94 (38.5)	
SAO (*n*, %)	93 (39.4)	36 (14.8)	
Unclassified (*n*, %)	27 (11.4)	20 (8.2)	
Recanalized therapy (*n*, %)	60 (25.4)	91 (37.2)	0.005
TSH (mU/L)	1.97 (1.29–3.22)	1.29 (0.73–2.51)	<0.001
FT3 (pmol/L)	4.04 ± 0.68	3.53 ± 0.70	<0.001
FT4 (pmol/L)	16.05 ± 2.55	16.64 ± 2.69	0.014

BMI: body mass index; AF: atrial ﬁbrillation; DM: diabetes mellitus; CHD: coronary heart disease; HF: heart failure; eGFR: estimated glomerular filtration rate; TG: triglyceride; TC: total cholesterol; HDL-C: high-density lipoprotein cholesterol; Glu: glucose; NIHSS: National Institutes of Health Stroke Scale; TOAST: Trial of Org 10172 in Acute Stroke Treatment; LAA: large-artery atherosclerosis; CE: cardioembolism; SAO: small-artery occlusion; TSH: thyroid-stimulating hormone; FT3: free triiodothyronine; FT4: free thyroxine.

**Table 2:** Multiple logistic regression analysis of predictors for poor outcomes.

Variables	OR	95% CI	*P* value
Age	1.023	1.001–1.047	0.044
Women	1.162	0.657–2.055	0.605
CHD/HF	1.116	0.618–2.015	0.716
AF	0.952	0.418–2.169	0.906
Hemoglobin	1.004	0.989–1.019	0.615
WBC	1.052	0.968–1.143	0.232
ALB	0.969	0.910–1.031	0.322
eGFR	1.001	0.987–1.015	0.873
TG	1.075	0.897–1.288	0.436
TC	1.166	0.881–1.543	0.283
NIHSS	1.286	1.213–1.363	<0.001
No recanalized therapy	3.527	1.808–6.880	<0.001
TSH	1.023	0.871–1.203	0.779
FT3	0.561	0.375–0.841	0.005
FT4	1.094	0.990–1.208	0.078
TOAST classification			
LAA	1	Reference	Reference
CE	0.709	0.306–1.643	0.422
SAO	0.700	0.368–1.332	0.278
Unclassified	0.568	0.236–1.371	0.209

**Table 3:** Odds ratios for poor outcomes according to the quartiles of FT3.

FT3 quartiles	Model 1		Mode 2	
	OR (95% CI)	*P* value	OR (95% CI)	*P* value
Q1 (<3.26 pmol/L)	4.63 (2.33–9.02)	<0.001	2.56 (1.15–5.69)	0.021
Q2 (3.26–3.79 pmol/L)	2.42 (1.35–4.35)	0.003	1.88 (0.96–3.71)	0.066
Q3 (3.79–4.26 pmol/L)	1.24 (0.69–2.23)	0.465	0.95 (0.48–1.87)	0.885
Q4 (>4.26 pmol/L)	1 (reference)	—	1 (reference)	—

Model 1 was adjusted for sex, AF, CHD/HF, hemoglobin, WBC, ALB, eGFR, TSH, TG, TC, FT4, and TOAST classification; model 2 was additionally adjusted for variables of age, NIHSS score, and recanalized therapy on the basis of model 1.

## Data Availability

The data that support the findings of this study are available from the corresponding author upon reasonable request.
